# Apert Syndrome: A Case Report

**DOI:** 10.5005/jp-journals-10005-1166

**Published:** 2012-12-05

**Authors:** Saba Khan, Laxmikanth Chatra, Prashanth Shenai, KM Veena

**Affiliations:** Postgraduate Student, Department of Oral Medicine and Radiology Yenepoya Dental College, Yenepoya University, Deralakatte, Mangalore-575018, Karnataka, India, e-mail: dr.sabakhan23@gmail.com; Senior Professor and Head, Department of Oral Medicine and Radiology Yenepoya Dental College, Yenepoya University, Mangalore, Karnataka India; Senior Professor, Department of Oral Medicine and Radiology Yenepoya Dental College, Yenepoya University, Mangalore, Karnataka India; Professor, Department of Oral Medicine and Radiology, Yenepoya Dental College, Yenepoya University, Mangalore, Karnataka, India

**Keywords:** Apert’s, Symmetric, Syndactyly, Craniosynostosis, Acrocephalosyndactylia, Midface hypoplasia

## Abstract

Apert syndrome (acrocephalosyndactyly) is a rare congenital disorder characterized by craniosynostosis, midfacial malformation and symmetrical syndactyly. We present a 10-month-old infant having all the features of classical Apert syndrome.

**How to cite this article:** Khan S, Chatra L, Shenai P, Veena KM. Apert Syndrome: A Case Report. Int J Clin Pediatr Dent 2012; 5(3):203-206.

## INTRODUCTION

Apert’s syndrome was described by Wheaton in 1894. In 1906, Dr Eugene Charles Apert published a summary on nine cases. Aperts syndrome or acrocephalosyndactylia is a developmental malformation characterized by craniosynostosis, a cone-shaped calvarium, midface hypoplasia, pharyngeal attenuation, ocular manifestations and syndactyly of hands and feets. According to Cohen, 15 out of 10 lakh live births are of Apert syndrome.^1^

## CASE REPORT

A 9-month-old boy presented with complaints of symmetric syndactyly of both hands and feet, abnormal head shape. Both the parents were normal and in third decade of life. He was the second child from a nonconsanguineous marriage and had one sibling who was normal and mother had a normal delivery with no history of trauma, infection, drug use during the term. No family history of similar complaints or any other congenital abnormality was reported.

**Fig. 1:**
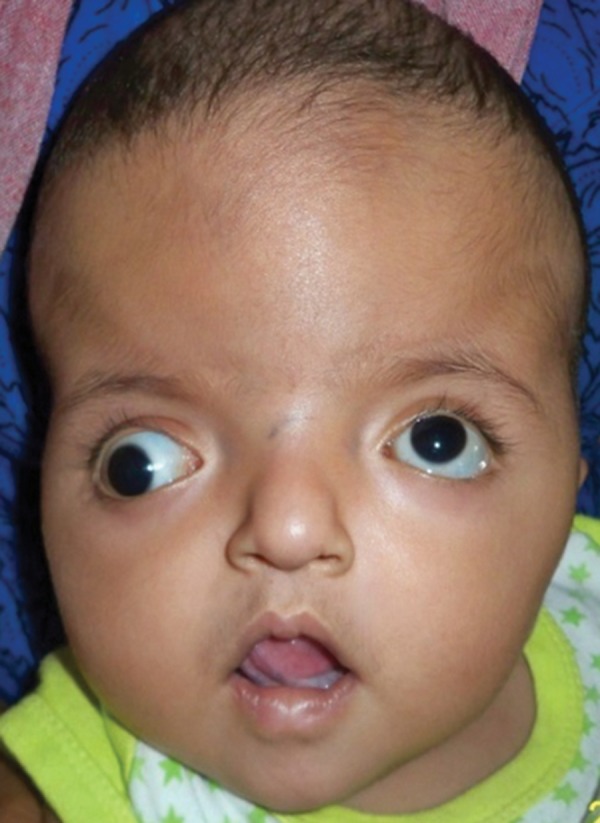
The infant with a turribrachycephalic (tall and decreased AP dimension) skull, frontal bossing, hypertelorism, depressed nasal bridge, antimongoloid slant of the eyes and midface deficiency

**Fig. 2:**
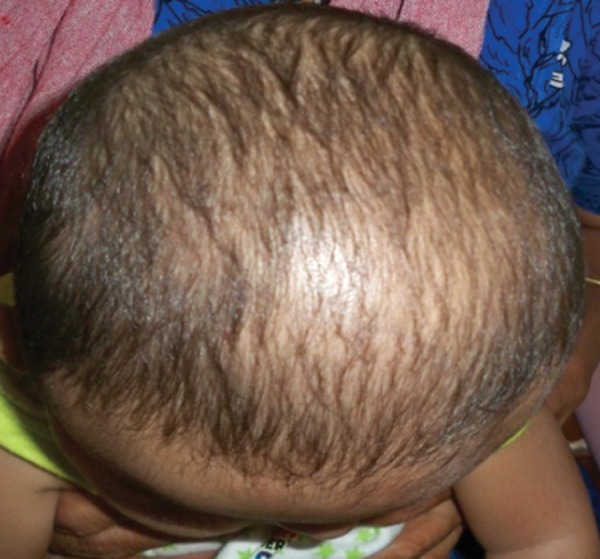
Dome-shaped protruberance in anterior parietal region and increased height of the skull

**Figs 3A and B:**
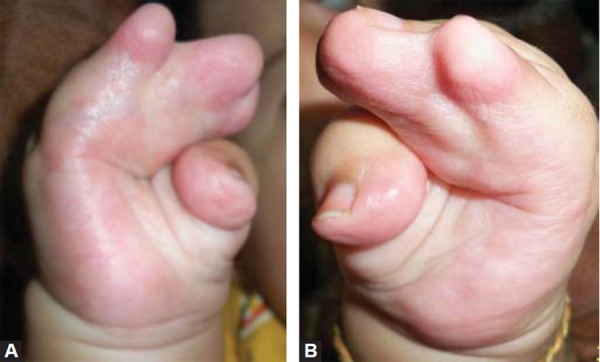
Bilateral symmetrical syndactyly with complete fusion of all the five digits of both hands with inwardly placed thumb

**Fig. 4:**
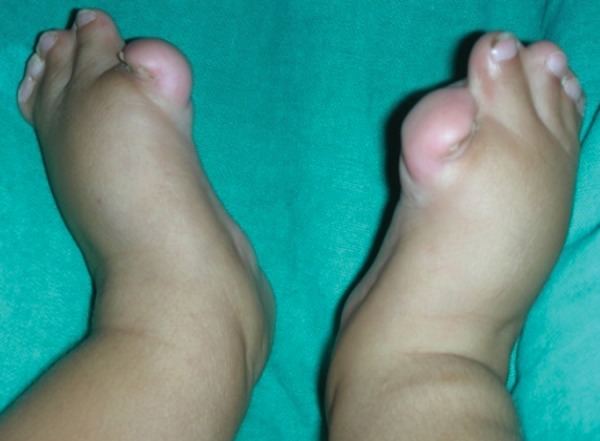
Bilateral symmetrical syndactyly of both the feets with deformation of the great toes

**Fig. 5:**
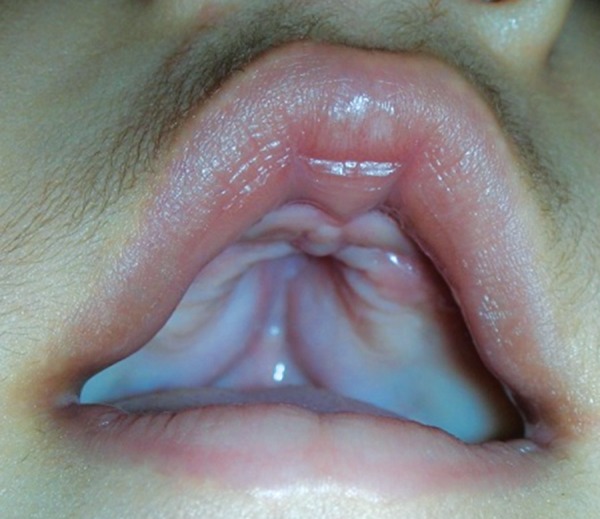
Deficient premaxilla, V-shaped maxillary arch, pseudocleft, cross bow-shaped lips

Examination revealed abnormal turribrachycephalic head contour (tall and AP shortened), flat occiput and a protuberant frontal region. Ocular proptosis, strabism, hypertelorism, down sliding lateral palpebral fissures were present. He had depressed nasal bridge and a thick nose with a bulbous tip and cross bow-shaped lips. He had midfacial deficiency with hypoplastic and retruded maxilla (Figs 1 and 2). Bilateral symmetrical syndactyly with complete fusion of all the five digits of both hands with inwardly placed thumb was present, also syndactyly was present with both feet with deformation of the great toe. The fused fingers and toes had separate nails (Figs 3A to 4). Intraorally, there was absence of teeth, V-shaped maxillary arch and a pseudocleft palate (Fig. 5). There was no other apparent congenital malformation, and systemic examination revealed no other abnormality.

**Fig. 6:**
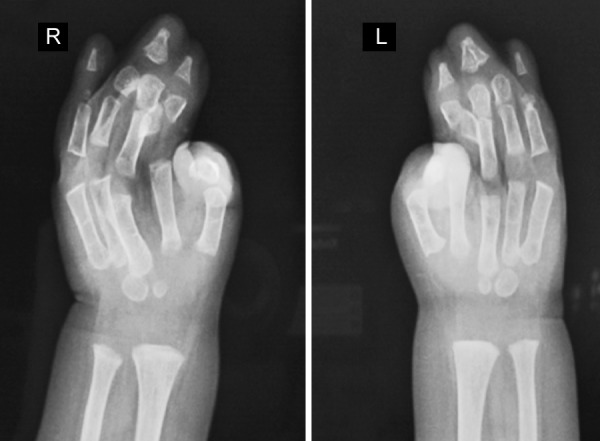
Hand-wrist radiograph showing soft tissue syndactyly of all the digits and synostosis involving phalanges of second, third and fourth digits and metacarpels of both the hands

**Fig. 7:**
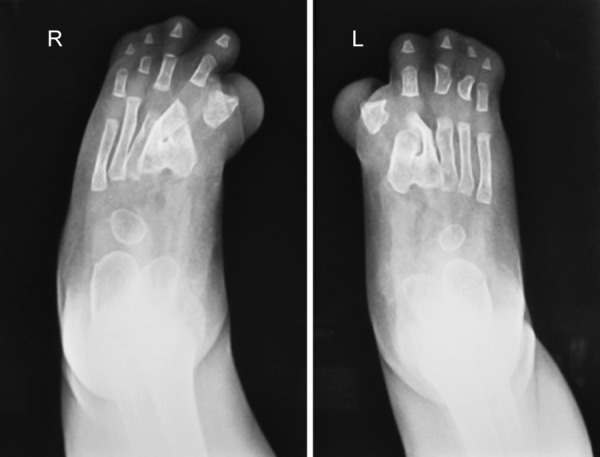
Soft tissue fusion of all the digits and synostosis of the metacarpals of both the feet with deformed great toes

**Fig. 8:**
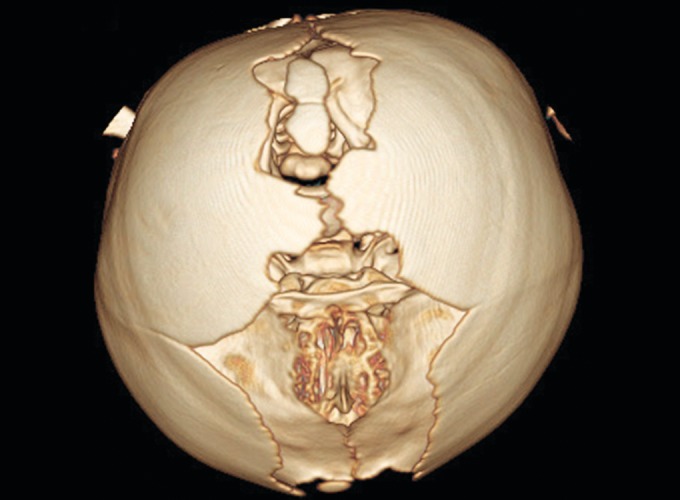
Three-dimensional CT showing a midline defect extending from glabella to posterior fontanelle with abnormally wide anterior and posterior fontanelle

**Fig. 9:**
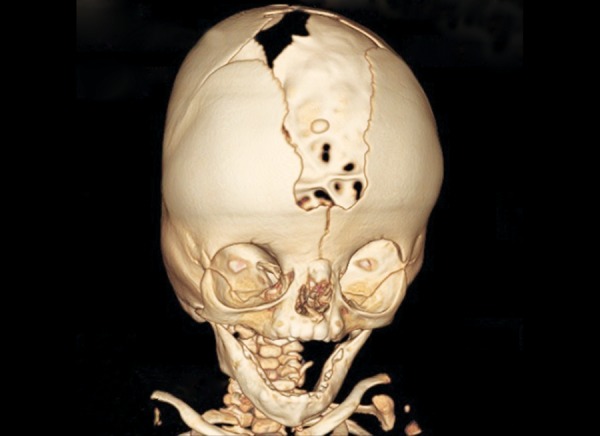
Bilateral symmetric synostosis of coronal and lambdoid sutures

Radiographs of both hands and feet showed soft tissue syndactyly of all the digits and synostosis involving phalanges of second, third and fourth digits of both the hands and the metacorpals of both the hands and feet with a deformed great toe (Figs 6 and 7). Anterioposterior skull radiographs revealed fused coronal sutures, turribrachycephalic skull contour, bitemporal widening, hypertelorism and increased convolutional markings. Three-dimensional CT reconstructions in a superoinferior view showed a midline defect extending from glabella to posterior fontanelle with abnormally wide anterior and posterior fontanelle (Fig. 8). Bilateral symmetric synostosis of coronal and lamdoid sutures was also present (Fig. 9). Axial sections at level of plexus choroideus showed agenesis of corpus callosum. All findings were diagnostic of Apert syndrome.

## DISCUSSION

Apert syndrome is a autosomal dominant disorder caused due to the mutation of fibroblast growth factor recptor-2 (FGFR-2) on chromosome 10q. Suture progenitor cells with mutated FGFR-2 cannot transduce signals from extracellular FGF, as a result they do not produce the fibrous material required for normal calvarial sutures.^2^ The majority of cases are sporadic, resulting from new mutations with a paternal age effect. The prodromal characteristics of typical turribrachycephalic head shape is early craniosynostosis of coronal sutures and agenesis of saggital and metopic sutures which results as a wide defect extending from glabella to posterior fontanelle. Also the spheno-occipital and sphenoethmoidal synchondrosis and early fusion of frontoethmoidal suture causes a shortened anterior and posterior cranial base with reduction in pharyngeal height. Premature fusion of sutures with continued brain growth can lead to increased intracranial pressure which can be seen as increased convolutional markings on skull radiographs. This inturn results in hypoplastic midface and a vertically accentuated craniofacial complex. Ocular proptosis, down slanting of lateral canthus and palpebral fissures (antimongoloid slant), hypertelorism are present due to shortening of the bony orbit. There is depressed nasal bridge with deviated nasal septum. The maxilla is hypoplastic and retropositioned. The lips are bow shaped and often unable to form a lip seal.^3^

Syndactylia or webbing of fingers causes immobility of fingers due to ossification of interphalangeal joints due to segmentation of embryonic phalanges. Involvement of the first or fifth digits in this bony mass is variable. There can be a similar deformity involving the foot (mitten hand and sock foot).^4^

Intraorally high arched and saggitally narrow palate seen with lateral palatal swellings with prominent central fissure and mostly presents with a pseudopalate. The maxillary arch is V shaped and saggitally narrow. Severe dental crowding, delayed tooth eruption, thick gingiva are common feature. Posterior slanting maxilla can give rise to class III malocclusion later in life.^5^ Central nervous system abnormalities (megalocephaly, pyramidal tract abnormalities), skeletal abnormalities (limited mobility of glenohumeral joint, elbow joint, etc.), cardiovascular, genitourinary and gastrointestinal, mental retardation, visual and hearing, speech defects have also been recorded. The literature also reports skin manifestations in Apert syndrome, such as acne, hyperhydrosis, hypopigmentation and hyperkeratosis of plantar surfaces.^3^

According to the literature, Apert’s and Crouzon’s syndrome seem to be the same syndrome, with the exception of syndactyly of hands and feet in Apert’s syndrome. Cleft or pseudocleft palate is a frequent finding in Apert’s syndrome, whereas these traits are extremely rare in Crouzon’s syndrome.^6^

The dentist is capable of recognizing the genetic disorders pertaining to orofacial structures. The treatment of apert syndrome begins at birth and a multidisciplinary approach is required arrive at a collaborative corrective plan for the deficiencies. Craniectomy is often performed during 6 months of age to treat the craniosynostosis. Corrective surgery for syndactyly is done in first year of life and completed by 3 to 4 years of age. Cosmetic correction for midface deficiency and pseudocleft is at 4 to 6 years age. Orthodontic and orthognathic surgery is performed after eruption of permanent dentition and completion of growth. Nonsurgical manipulation of Apert syndrome may be a possibility in the future, for example by using selective inhibitors of the FGFR-kinase domain.**^7^** Genetic counseling is an important factor as recurrence risk for an affected individual to have an affected offspring is 50%.
